# Assessment of Sarcopenia Using Rectus Femoris Ultrasound in Emergency Patients—A Cross-Sectional Study

**DOI:** 10.3390/jcm14113932

**Published:** 2025-06-03

**Authors:** Francisco Javier García-Sánchez, Victoria Emilia Souviron-Dixon, Fernando Roque-Rojas, Natalia Mudarra-García

**Affiliations:** 1 Emergency Room Service, Hospital Universitario Infanta Cristina, Instituto de Investigación Sanitaria Hospital Puerta de Hierro Segovia Arana (IDIPHISA), 28981 Madrid, Spain; victoriaemilia.souviron@salud.madrid.org (V.E.S.-D.); fernando.roque@salud.madrid.org (F.R.-R.); 2 Medical Department, Faculty of Medicine, University Complutense of Madrid, 28040 Madrid, Spain; 3 Nursing Department, Faculty of Medicine, University CEU San Pablo, 28003 Madrid, Spain; 4 Instituto Ramón y Cajal de Investigación Sanitaria (IRYCIS), 28034 Madrid, Spain; nmudarra@enf.ucm.es; 5 Nursing Department, Faculty of Nurse, Phisiotherapy and Podology, University Complutense of Madrid, 28040 Madrid, Spain

**Keywords:** sarcopenia, muscle ultrasound, body composition, rectus femoris

## Abstract

**Background**: Sarcopenia is a progressive muscle disorder commonly associated with aging and chronic diseases. It has been linked to worse clinical outcomes and increased vulnerability during acute illness. However, its prevalence in emergency department (ED) populations remains underexplored. This study aimed to evaluate the presence of sarcopenia among ED patients using ultrasound, determine its relationship with underlying comorbidities, and assess its association with in-hospital complications. **Methods**: We conducted a prospective, observational, cross-sectional study at the Infanta Cristina University Hospital (Madrid, Spain) from January to May 2024. A total of 150 patients aged 18 years and older who presented to the ED were assessed for sarcopenia using rectus femoris ultrasound. Sociodemographic, clinical, and laboratory variables were collected. A multivariate logistic regression model was used to identify independent predictors of in-hospital complications. Patients were followed for 30 days to evaluate outcomes. Comparisons were made between diagnostic groups and sarcopenia indices. **Results**: The mean age of the cohort was 70.7 years (SD 18.15), and 52% were male. Neurological diseases were associated with the highest degree of sarcopenia (mean Y-axis: 0.93 cm), followed by digestive (1.05 cm), hematological (1.05 cm), and cardiovascular diseases (1.08 cm). Patients who developed in-hospital complications had lower mean muscle thickness values compared to those without complications (1.08 cm vs. 1.24 cm; *p* < 0.05). Sarcopenia was significantly correlated with the presence of comorbidities and poor clinical outcomes. **Conclusions**: These findings support the integration of sarcopenia screening protocols into emergency care and highlight the need for studies exploring early nutritional or rehabilitation interventions targeted at high-risk patients.

## 1. Introduction

Sarcopenia is a skeletal muscle disorder characterized by the progressive loss of muscle mass, strength, and function [1,2]. The European Working Group on Sarcopenia in Older People (EWGSOP2) has established a diagnostic algorithm that includes the assessment of muscle strength, mass, and quality [3]. Classified as a disease in the ICD-10-CM [4], sarcopenia is common among older adults due to the progressive decline in skeletal muscle tissue beginning around the age of 40, increasing the risk of frailty and functional dependence [5,6].

Sarcopenia is associated with multiple comorbidities, including cancer [7], obesity [8,9], and renal and cardiovascular diseases [10]. Its prevalence is difficult to determine due to the wide range of diagnostic methods—such as the SARC-F questionnaire, BIA, MRI, CT, and DEXA [1]—which differ in accessibility, accuracy, and clinical utility [11,12]. Early detection of sarcopenia in emergency settings may offer a window of opportunity to initiate timely interventions, particularly in frail or comorbid patients [13].

Cardiovascular diseases such as heart failure and peripheral arterial disease may contribute to sarcopenia due to factors like oxidative stress and malnutrition [14]. Likewise, cardiac surgery can lead to muscle mass loss due to immobilization [10]. In respiratory conditions such as chronic obstructive pulmonary disease (COPD), dyspnea and chronic inflammation worsen sarcopenia [15]. There is also evidence of higher sarcopenia prevalence in patients with malignant digestive diseases [16] and in oncology patients with cachexia, a metabolic syndrome involving loss of muscle mass and body weight [17,18].

Trauma is also associated with sarcopenia, as appropriate nutritional support can reduce rehabilitation time in cases such as hip fractures [18]. Among metabolic disorders, type 2 diabetes mellitus (T2DM) contributes to sarcopenia through insulin resistance and lipid-induced muscle dysfunction [19].

Given the clinical relevance of this condition, the present study aimed to evaluate the relationship between sarcopenia and various pathologies in patients admitted to the emergency department, to identify which diseases are associated with higher sarcopenia indices, and to determine the related in-hospital complications.

We hypothesized that lower rectus femoris muscle thickness, indicative of sarcopenia, would be associated with a higher prevalence of comorbidities and increased risk of in-hospital complications among emergency department patients.

## 2. Materials and Methods

### 2.1. Study Design

This was an observational, prospective, and cross-sectional study conducted at the Infanta Cristina University Hospital in Parla (Madrid, Spain). Sarcopenia was assessed by ultrasound, performed under the supervision of a liaison nurse, in all patients who presented to the emergency department. The results were compared across the different diagnosed pathologies.

This study was approved by the Ethics and Research Committee of the Puerta de Hierro University Hospital (ACT 245.23, 24 November 2023).

The reporting of this observational study followed the STROBE (Strengthening the Reporting of Observational Studies in Epidemiology) guidelines.

### 2.2. Study Population

The nutritional ultrasound program for the diagnosis of sarcopenia was implemented at the hospital in 2021. All patients presenting to the emergency department were evaluated using ultrasound, except for those who did not meet the inclusion criteria. All eligible patients who met the inclusion criteria were included and completed the full study protocol without loss to follow-up.

#### 2.2.1. Inclusion Criteria

The study included all patients aged 18 years or older who presented to the Emergency Department of Infanta Cristina University Hospital between January and May 2023. To be eligible, patients had to be in stable clinical condition, allowing for ultrasound evaluation during their emergency department stay—either during prolonged observation or prior to hospital admission. Additionally, patients were required to have sufficient cognitive capacity to understand the information provided about the procedure and to give verbal consent for the assessment.

#### 2.2.2. Exclusion Criteria

Patients were excluded from the study if, at the time of evaluation, they were in cardiac arrest (resuscitation bay), exhibited hemodynamic instability that precluded immediate ultrasound assessment, presented with an acute psychiatric condition that impaired cooperation, or had clinical presentations classified as high-resolution cases, where the short duration of stay in the emergency department did not allow for completion of the sarcopenia measurement protocol.

### 2.3. Sample Size

Assuming a 15% loss rate, a 95% confidence level, a 3% margin of error, and a 5% estimated proportion, an appropriate sample size of 150 participants was calculated.

### 2.4. Studied Variables

The following sociodemographic and clinical variables were collected: sex (male, female), age, nutritional ultrasound measurements of the rectus femoris (Y-axis, X-axis, and cross-sectional area), antidiabetic medication (metformin, sulfonylureas, sodium–glucose co-transporter 2 (iSGLT2) inhibitors, thiazolidinediones, dipeptidyl peptidase-4 (iDPP-4) inhibitors, insulin, glucagon-like peptide-1 (GLP-1Ra) receptor agonists, and their respective combinations), reason for admission or observation (cardiovascular, respiratory, endocrine-metabolic, digestive, oncological, neurological, orthopedic, urological, hematological), and personal medical history (yes/no) of cardiovascular disease, digestive disease, respiratory disease, urological disease, hematological disease, neurological disease, diabetes mellitus, and active cancer.

The clinical outcomes assessed included the following: hospital admission (yes/no), C-reactive protein (CRP), lymphocyte count, total protein, albumin, development of complications (yes/no), type of complication (infectious, respiratory, cardiovascular), and in-hospital mortality (yes/no).

Complications were defined as new events during hospitalization, including respiratory infections, cardiovascular events (e.g., arrhythmias, acute coronary syndromes), and other nosocomial infections, as documented in the electronic medical records.

### 2.5. Intervention

All patients included in the study underwent a nutritional ultrasound assessment aimed at estimating their degree of sarcopenia. Prior to the procedure, each patient’s capacity to understand the purpose, implications, and rationale for their inclusion in the program was evaluated to ensure adequate informed cooperation.

The ultrasound examination was performed during the emergency department stay, either when the patient required prolonged observation or had a pending hospital admission. At the same time, relevant clinical data were collected, including the reason for admission, personal medical history, and current medications.

The muscle ultrasound was conducted using a Mindray Z50® ultrasound system, Nanshan, Shenzhen, China equipped with a linear transducer, optimized for high-resolution imaging of superficial skeletal muscle tissue. The patient was placed in a supine position with the lower limbs relaxed to minimize muscle tension.

**Figure 1:**
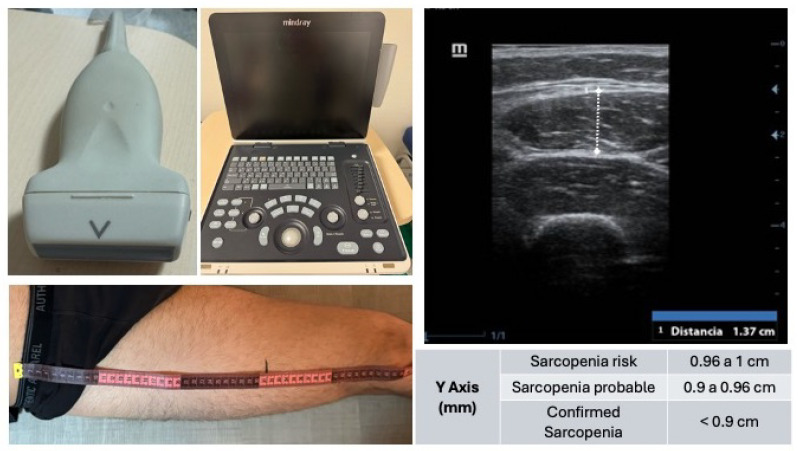
Ultrasound assessment of the rectus femoris muscle for sarcopenia screening. Shown (**top left**) is the linear probe (10–12 MHz) used for image acquisition, alongside the portable ultrasound system Mindray Z60 (**center**). The ultrasound image (**top right**) corresponds to a transverse view at the distal third of the thigh. Identified from superficial to deep: ultrasound gel, skin, subcutaneous tissue, vastus lateralis muscle, rectus femoris muscle, and femur. The bottom left image depicts the anatomical landmarking process for measurement based on the distance between the anterior superior iliac spine and the upper edge of the patella. Reference values for the Y-axis measurement of the rectus femoris are included and used to classify sarcopenia risk and diagnosis.

The anatomical reference points for the measurement were the anterior superior iliac spine (ASIS) and the superior border of the patella. The total distance between these landmarks was measured, and the probe was positioned at the junction of the distal third of this segment, a location providing optimal visualization of the rectus femoris muscle (Figure 1).

After applying conductive gel to the skin, the transducer was placed perpendicularly to the muscle fibers in the transverse (short-axis) plane. Great care was taken to avoid any probe angulation, as oblique positioning can lead to erroneous measurements. Once the muscle architecture was clearly identified—typically including the skin, subcutaneous tissue, vastus lateralis, rectus femoris, and femoral bone interface—the image was frozen, and the anteroposterior thickness of the rectus femoris (Y-axis) was measured, along with other optional parameters (X-axis, cross-sectional area).

Following the ultrasound assessment, relevant laboratory values (e.g., C-reactive protein, lymphocyte count, total protein, and albumin levels) were recorded from the electronic health records. Additionally, at one month post-admission, a retrospective review of the patient’s clinical course was conducted to identify any in-hospital complications (e.g., infections, cardiovascular, or respiratory events) and to register mortality (exitus) where applicable.

### 2.6. Statistical Analysis

Statistical analysis was performed using SPSS software (version 29; IBM, Armonk, NY, USA). Descriptive statistics were calculated for all variables. Categorical variables were presented as frequencies and percentages, and continuous variables were expressed as means and standard deviations.

To assess the independent association between rectus femoris muscle thickness and the development of in-hospital complications, a multivariate logistic regression model was constructed. The model included the Y-axis muscle measurement as the primary predictor and adjusted for potential confounders, including age, sex, and the presence of cardiovascular, digestive, neurological, hematological, and respiratory comorbidities. Statistical significance was set at *p* < 0.05.

## 3. Results

### 3.1. General Characteristics of the Population

A total of 150 patients were included. Among the participants, 52.00% were male. The mean age was 70.70 years (SD 18.15). Thirty percent of participants were diabetic, of whom twenty-four percent were treated with oral antidiabetic agents, with metformin being the most commonly used (8.70%). Additionally, 30.70% of the patients had a cancer diagnosis. The most frequent comorbidities were cardiovascular diseases, present in 60% of the patients (Table 1 and Table 2).

### 3.2. Clinical Outcomes One Month After Admission

Among the 150 patients presenting to the emergency department at HUIC, 61.3% required hospital admission, and 42% of those developed complications. The most common complications were infectious in nature (20%). The number of patients who died during hospitalization was seven (4.7%) (Table 3).

### 3.3. Relationship Between Sarcopenia and Comorbidities

The most significant sarcopenia values were observed in patients with neurological conditions, with a mean rectus femoris Y-axis measurement of 0.93 cm (Table 4 and Table 5).

### 3.4. Relationship Between Sarcopenia and Complications

Among the patients included in the sample, 42% experienced complications during hospitalization. The sarcopenia index was notably lower in patients who developed complications compared to those who did not (1.0786 (SD 0.34) vs. 1.2414 (SD 0.38)) (Table 6).

### 3.5. Multivariate Analysis

A multivariate logistic regression analysis was performed to identify independent predictors of in-hospital complications. The model included rectus femoris muscle thickness (Y-axis), age, sex, and comorbidities (cardiovascular, digestive, neurological, hematological, and respiratory).

A lower rectus femoris muscle thickness was significantly associated with an increased risk of complications (OR: 0.21, 95% CI: 0.06–0.79; *p* = 0.021). Male sex was marginally associated with higher risk (OR: 2.15, 95% CI: 1.00–4.61; *p* = 0.050), while age and comorbidities did not reach statistical significance in the adjusted model. These findings highlight the prognostic role of sarcopenia in emergency care settings (Table 7).

## 4. Discussion

This study describes the sarcopenia outcomes of patients who presented to the emergency department, as well as the prevalence of the condition according to different underlying diseases. Sarcopenia has often been reported as a comorbidity of cardiac conditions. A literature review identified sarcopenia as one of the most important factors contributing to impaired physical function and the progression of cardiovascular disease [5]. In the present study, cardiovascular disease was the most frequent reason for emergency consultation (20.70%) and also the most common comorbidity, with 91 out of 150 patients having a history of cardiovascular disease.

Stroke ranked as the second leading cause of death in Spain during the first half of 2023, following ischemic heart disease [20]. Sarcopenia has been linked to such conditions due to its role in capillary alterations in skeletal muscle tissue and its association with neuronal degeneration [21]. In ischemic stroke, cerebral injury leads to synaptic remodeling, including alterations in motor neuron innervation, which increases the likelihood of developing sarcopenia. Among the 150 participants, neurological pathology showed the highest degree of sarcopenia, with a mean Y-axis measurement of 0.93 cm in the rectus femoris. However, this group was relatively small, as only 23 out of 150 patients presented with neurological symptoms. Most were ischemic stroke survivors with one or more cardiovascular risk factors, though other clinical conditions, such as epilepsy, were also represented.

A descriptive observational study of 303 patients with digestive diseases showed that 32.00% had a diagnosis of sarcopenia. Sarcopenia prevalence was reported as 22.20% in gastrointestinal disease, 36.60% in biliary–pancreatic disease, and 36.90% in hepatic disease [16]. A literature review further concluded that early-onset Crohn’s disease and advanced-age ulcerative colitis were associated with a higher risk of sarcopenia due to the malabsorption and inflammation characteristic of these conditions [22]. The present study supports this evidence, showing that patients with digestive diseases had the second-highest prevalence of sarcopenia, with a mean Y-axis measurement of 1.05 cm.

Regarding hematological conditions, a direct relationship was also observed with sarcopenia. A cross-sectional study indicated that low hematocrit, decreased hemoglobin levels—as in anemia—and increased IL-6 were associated with a greater likelihood of sarcopenia [23]. Similarly, our findings placed hematological conditions as the third leading group in terms of sarcopenia prevalence, with a mean of 1.05 cm.

Finally, patients with respiratory conditions in this study exhibited the lowest degrees of sarcopenia, as there were no substantial numerical differences in Y-axis means between patients with or without a respiratory history. However, certain respiratory conditions, such as pneumonia, are associated with inflammation that promotes muscle atrophy via a pro-inflammatory cytokine cascade [21]. It is noteworthy that the majority of complications observed after hospital admission in this study were infectious respiratory events, often requiring extended hospital stays and contributing to physical decline and muscle mass loss. Emerging literature has also highlighted the relevance of sarcopenia in infectious diseases such as COVID-19, where low muscle mass has been associated with worse outcomes [24].

Despite the variation in the frequency of diagnoses and Y-axis measurements, most patients with any of the comorbidities listed above showed some degree of sarcopenia, with Y-axis means generally around 1.0–1.1 cm. The results of this study demonstrate that neurological pathology was associated with the lowest muscle thickness and highest sarcopenia index, with a mean of 0.93 cm, followed by digestive, hematological, and cardiac conditions with means of 1.05 cm, 1.05 cm, and 1.08 cm, respectively.

The multivariate analysis confirmed that reduced rectus femoris thickness, as a surrogate marker of sarcopenia, was independently associated with the development of in-hospital complications. This reinforces prior evidence suggesting that muscle mass serves as a marker of physiological reserve and vulnerability. Interestingly, none of the specific comorbidities reached significance in the adjusted model, which underscores the potential value of incorporating muscle ultrasound into routine risk stratification.

**Figure A1:**
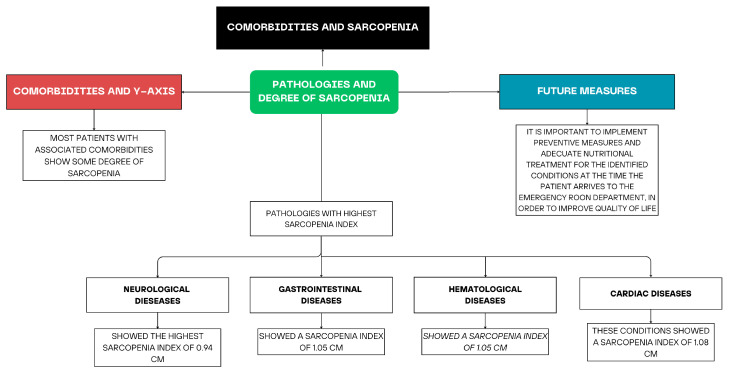
Infographic summarizing the relationship between sarcopenia and comorbidities, highlighting the pathologies with the highest sarcopenia index (Y-axis) and emphasizing the importance of early preventive and nutritional interventions in emergency settings.

The marginal association between male sex and complications aligns with some literature suggesting sex-related differences in inflammatory and metabolic response. Further studies are needed to explore these gender-based variations (Figure A1).

## 5. Conclusions

- Sarcopenia is highly prevalent among ED patients, especially those with neurological and digestive diseases. Muscle ultrasound is a feasible screening tool in acute care settings and may aid in the early identification of patients at higher risk of adverse outcomes. Lower rectus femoris thickness was independently associated with the development of complications, supporting its role as a prognostic marker in emergency care.
- Neurological conditions showed the highest association with severe sarcopenia, followed by digestive, hematological, and cardiovascular diseases, based on rectus femoris muscle ultrasound measurements.
- Muscle ultrasound is a feasible, non-invasive screening tool in the emergency setting, offering an objective evaluation of muscle mass that may help stratify clinical risk.
- A higher degree of sarcopenia was associated with an increased risk of in-hospital complications, supporting its role as an early marker of frailty and clinical deterioration.
- The assessment of sarcopenia should be integrated into emergency care protocols, especially for older adults and patients with neurological, digestive, or hematological conditions.

Further research is warranted to develop targeted nutritional and physical interventions that can improve clinical outcomes beginning from the emergency department phase.

## 6. Study Limitations

- This study was conducted at a single hospital center, which may limit the generalizability of the findings to other populations or clinical settings.
- The cross-sectional observational design does not allow for the establishment of causal relationships between sarcopenia and clinical outcomes.
- Although the sample size was adequately estimated, it may not have been large enough to detect subtle differences between certain clinical subgroups.
- Data on baseline functional status, physical activity levels, and nutritional intake were not collected, despite being relevant factors in the development of sarcopenia.

Notably, there were no losses to follow-up or exclusions after enrollment. All 150 participants were successfully evaluated at all stages of the study, including ultrasound assessment and 30-day outcome follow-up. This complete case analysis enhances the methodological robustness of the study.

## 7. Future Research Directions

- Expand the study to multiple centers with varying healthcare characteristics and patient populations to validate and compare findings.
- Design longitudinal studies to evaluate the progression of sarcopenia and its impact on medium- and long-term clinical outcomes.
- Assess the effectiveness of early nutritional and physical interventions initiated in the emergency department to prevent functional decline.
- Include additional variables such as functional assessment, nutritional status, systemic inflammation, and quality of life measures.
- Investigate the use of muscle ultrasound as a dynamic marker for therapeutic response monitoring.

## Figures and Tables

**Table 1:** General characteristics of the study population (*n* = 150).

Variables	Values
Age (mean ± SD)	70.74 ± 18.16
Y-axis (mean ± SD)	1.17 ± 0.38
X-axis (mean ± SD)	3.49 ± 0.79
Area (mean ± SD)	3.45 ± 1.69
Sex—Male	78 (52.00%)
Sex—Female	72.00 (48.00%)
Diabetic—Yes	45.00 (30.00%)
Diabetic—No	105.00 (70.00%)
Patients on Oral Antidiabetics	36.00 (24%)
Cardiovascular history—Yes	91.00 (60.7%)
Cardiovascular history—No	59.00 (39.3%)
Oncological—Yes	46.00 (30.70%)
Oncological—No	104.00 (69.30%)
Digestive history—Yes	43.00 (28.70%)
Digestive history—No	107.00 (71.30%)
Respiratory history—Yes	32.00 (21.30%)
Respiratory history—No	118.00 (78.70%)
Urological history—Yes	30.00 (20.00%)
Urological history—No	120.00 (80.00%)
Hematological history—Yes	27.00 (18.00%)
Hematological history—No	123.00 (82.00%)
Neurological history—Yes	23. (15.30%)
Neurological history—No	127.00 (84.70%)

**Table 2:** Comparison of rectus femoris Y-axis thickness according to the presence or absence of comorbidities.

Comorbidity	With Comorbidity (Mean ± SD)	Without Comorbidity (Mean ± SD)	*p*-Value
Cardiovascular	1.08 ± 0.33	1.32 ± 0.41	<0.001
Respiratory	1.20 ± 0.42	1.17 ± 0.37	0.691
Digestive	1.05 ± 0.28	1.22 ± 0.40	0.009
Neurological	0.93 ± 0.29	1.22 ± 0.38	0.001
Hematological	1.05 ± 0.38	1.20 ± 0.39	0.061
Urological	1.15 ± 0.31	1.18 ± 0.40	0.713
Oncological	1.05 ± 0.29	1.23 ± 0.4061	0.011

**Table 3:** Clinical characteristics one month after hospital admission.

Variables	Values
Hospital admission—Yes	92.00 (61.30%)
Hospital admission—No	58.00 (38.70%)
Complications—Yes	63.00 (42.00%)
Complications—No	87.00 (58.00%)
Type of complication—Infectious	30.00 (20.00%)
Type of complication—Respiratory	16.00 (10.70%)
Type of complication—Cardiovascular	15.00 (10.00%)
In-hospital mortality—Yes	7.00 (4.70%)
In-hospital mortality—No	143.00 (95.30%)

**Table 4:** Clinical characteristics at emergency department presentation.

Variables	Values
Admission reason—Cardiovascular	31.00 (20.70%)
Admission reason—Respiratory	28.00 (18.70%)
Admission reason—Digestive	27.00 (18.00%)
Admission reason—Infectious	22.00 (14.70%)
Admission reason—Endocrine-metabolic	10.00 (6.70%)
Admission reason—Neurological	10.00 (6.70%)
Admission reason—Orthopedic	8.00 (5.30%)
Admission reason—Urological	7.00 (4.70%)
Admission reason—Oncological	5.00 (3.30%)
Admission reason—Hematological	2.00 (1.30%)
CRP (mg/L)	50.90 ± 77.47
Lymphocytes (U/L)	1330.40 ± 847.54
Total proteins (g/dL)	9.40 ± 27.23
Albumin (g/dL)	3.18 ± 0.99

**Table 5:** Relationship between sarcopenia and personal medical history.

Comorbidity	Mean Y-Axis (cm)
Neurological	0.93
Digestive	1.05
Hematological	1.05
Cardiovascular	1.08
Respiratory	1.20

**Table 6:** Relationship between sarcopenia and complications.

Group	Mean Y-Axis (cm)	SD
With complications	1.09	0.35
Without complications	1.24	0.39

**Table 7:** Multivariate logistic regression model for in-hospital complications (*n* = 150).

	OR	SE	*p*	CI 2.5%	CI 97.5%
Intercept	1.574	1.482	0.759	0.086	28.749
Y-axis	0.213	0.671	0.021	0.057	0.793
Age	1.013	0.014	0.358	0.986	1.041
Gender	2.148	0.390	0.050	0.999	4.618
Cardiovascular disease	0.833	0.402	0.649	0.379	1.831
GE disease	0.572	0.404	0.166	0.259	1.262
Neurological disease	0.557	0.520	0.261	0.201	1.544
Hematological disease	1.590	0.463	0.316	0.642	3.941
Respiratory disease	0.848	0.434	0.705	0.363	1.985

## Data Availability

The original contributions presented in this study are included in the article. Further inquiries can be directed to the corresponding author.

## Abbreviations

The following abbreviations are used in this manuscript:

ASIS	Anterior superior iliac spine
BIA	Bioelectrical impedance analysis
COPD	Chronic obstructive pulmonary disease
CRP	C-reactive protein
CT	Computed tomography
DEXA	Dual-energy X-ray absorptiometry
EWGSOP2	European Working Group on Sarcopenia in Older People
GLP-1ra	Glucagon-like peptide-1 receptor agonist
DPP-IVi	Dipeptidyl peptidase-4 inhibitor
MRI	Magnetic resonance imaging
SD	Standard deviation
SGLT-2i	Sodium–glucose co-transporter inhibitor
T2DM	Type 2 diabetes mellitus
